# Artificial intelligence agents in orthopaedics: Concepts, capabilities and the road ahead

**DOI:** 10.1002/ksa.70109

**Published:** 2025-10-17

**Authors:** Felix C. Oettl, James Pruneski, Balint Zsidai, Yinan Yu, Ting Cong, Robert Feldt, Philipp W. Winkler, Michael T. Hirschmann, Kristian Samuelsson, ESSKA Artificial Intelligence Working Group

**Affiliations:** ^1^ Department of Orthopedic Surgery, Balgrist University Hospital University of Zürich Zurich Switzerland; ^2^ Department of Orthopaedic Surgery Tripler Army Medical Center Honolulu Hawaii USA; ^3^ Sahlgrenska Sports Medicine Center Gothenburg Sweden; ^4^ Department of Orthopaedics, Institute of Clinical Sciences, Sahlgrenska Academy University of Gothenburg Gothenburg Sweden; ^5^ Department of Orthopedics Skåne University Hospital Malmö/Lund Sweden; ^6^ Department of Computer Science and Engineering Chalmers University of Technology Gothenburg Sweden; ^7^ Department of Orthopaedic Surgery University of Pittsburgh Pittsburgh Pennsylvania USA; ^8^ Department for Orthopaedics and Traumatology, Kepler University Hospital GmbH Johannes Kepler University Linz Linz Austria; ^9^ Department of Orthopaedic Surgery and Traumatology Kantonsspital Baselland Bruderholz Switzerland; ^10^ University of Basel Basel Switzerland; ^11^ Department for Orthopaedics Sahlgrenska University Hospital Mölndal Sweden

**Keywords:** agentic AI, artificial intelligence, clinical decision support, medical ethics, multiagent systems, surgical workflow

## Abstract

Artificial intelligence (AI) is increasingly used in orthopaedics, yet current models are often limited to narrow, isolated tasks like analysing an X‐ray or predicting a single outcome. This paper introduces AI agents—a new class of AI systems designed to overcome these limitations. Unlike traditional AI, agents can autonomously manage complex, multistep processes that mirror the complete patient journey. They can coordinate tasks from initial diagnosis and surgical scheduling to postoperative monitoring and rehabilitation, acting as intelligent assistants for clinical teams. This review explains what distinguishes AI agents from conventional AI, explores their potential applications in orthopaedic practice—including perioperative workflow optimisation, research acceleration and intelligent physician support—and discusses the significant implementation and ethical challenges that must be addressed. For the orthopaedic surgeon, understanding AI agents is becoming essential, as these systems offer a transformative potential to enhance efficiency, improve patient outcomes and shape the future of clinical leadership in a technologically advancing field.

**Level of Evidence:**

Level V.

**Abbreviations**
AI: artificial intelligence
API: advanced programming interfaces
EHR: electronic health record
EMA: European Medicines Agency
FDA: Food and Drug Administration
HRL: hierarchical reinforcement learning
LLM: large language model
MAS: multiagent systems
ML: machine learning
NLP: natural language processing
RL: reinforcement learning

## INTRODUCTION

The use of artificial intelligence (AI) in orthopaedic practice and research has increased dramatically in recent years, with use cases to include image analysis [18, 24, 45] and outcome prediction [6, 23, 36]. Machine learning (ML), natural language processing (NLP) and deep learning have become valuable tools for gaining insight into musculoskeletal injury and recovery [7, 31, 37, 38, 55]. However, the integration of these technologies into routine practice has been slow [2, 5, 8], partly because current AI models are often fragmented, addressing single problems in isolation.

Orthopaedics presents unique clinical challenges that traditional AI struggles to address comprehensively. The longitudinal nature of musculoskeletal conditions requires continuous monitoring and adaptive treatment planning over extended timeframes. Furthermore, effective orthopaedic decision‐making relies on integrating multimodal data—including imaging, patient‐reported outcomes, clinical assessments and biomechanical measurements—which exceeds the capabilities of most single‐purpose AI models. This personalised approach, tailored to each patient's unique anatomy and recovery, demands more sophisticated technological solutions.

The emergence of AI agents represents a paradigm shift in healthcare technology. AI agents are software systems designed to perceive their environment, reason, make decisions and act autonomously to achieve specific goals [11, 50]. Unlike traditional AI models that execute predefined tasks, agents possess defining features like autonomy and adaptability [5, 11, 19, 22]. By harnessing ML, NLP and predictive analytics, they can emulate human cognitive functions, processing vast amounts of data to manage complex, goal‐oriented processes [11, 42]. A particularly powerful capability of AI agents is their potential to operate collaboratively in what has been called a ‘society of mind’ [54]. This orchestration of multiple specialised agents enables the handling of complex, multimodal data while maintaining transparency through natural language interfaces—characteristics especially valuable for the multifaceted decision‐making required in orthopaedics.

Outside of orthopaedics, AI agents have been used for drug discovery [26], designing novel viral nanobodies [46], outcome prediction [16] and even modelling hospital care [25]. Beyond healthcare, agentic AI is being utilised for finance, cybersecurity and manufacturing tasks as well [30, 52, 53]. Despite these advances in other domains, there remains a paucity of literature describing specific use cases for AI agents within orthopaedics, where their capabilities could address the field's unique longitudinal, multimodal and personalised care requirements.

Accordingly, the purpose of the current review was to examine the current state and near‐future potential of AI agents, in orthopaedics, providing a framework for understanding potential use‐cases, limitations and implementation pathways.

## FUNDAMENTALS OF AGENTIC AI

Agentic AI systems utilise a composite architecture with components that function in tandem. An agent typically uses a foundation model (often a large language model, or LLM) as its central reasoning engine. This is augmented with memory mechanisms to preserve contextual information and planning modules that decompose complex goals into executable subtasks. Interactions with external data sources are achieved by leveraging advanced programming interfaces (APIs), while self‐reflection mechanisms enable metacognitive evaluation and iterative improvement through experiential learning.

Concisely, the essential feature of contemporary agentic AI is the synchronous implementation of multiple components where the foundation model acts as a meta‐controller that orchestrates complex workflows through recursive self‐monitoring. Further characteristics that distinguish AI agents from conventional AI systems include autonomy, goal‐oriented capacity, adaptability and social ability [49].

While traditional ML models are often trained on large, labelled datasets using supervised learning to perform specific classification or prediction tasks [29], agentic AI systems are typically built on foundations such as reinforcement learning (RL) and NLP using LLMs [1]. With RL, agents learn to maximise rewards by interacting with their environment to achieve goals through trial‐and‐error. Traditional models in orthopaedics, such as those developed for detecting fractures from X‐rays [14, 27] or assisting in preoperative planning [40, 44, 51], represent isolated, task‐specific systems. In contrast, AI agents integrate such capabilities into broader, goal‐directed workflows. For example, an agent might use an image analysis model as a tool, then reason about the findings, access patient records, consult clinical guidelines, propose a treatment plan and initiate scheduling or documentation [11, 34]. This orchestration mirrors how medical professionals navigate patient care.

The distinction between agentic behaviour and agentic implementation is crucial for healthcare. Agentic behaviour refers to what the system does—the observable actions and capabilities of a system, including autonomous operation, goal pursuit, adaptation to changing circumstances, and varying degrees of proactiveness or reactiveness [11, 50]. This is the external manifestation of agency. A system might exhibit agentic behaviour even if its internal architecture isn′t strictly agent‐based [4, 11]. Agentic Implementation refers to how the system is built. Is it designed using agent‐oriented principles, potentially with explicit goals, beliefs, planning capabilities and learning mechanisms? Architectures can range from simple reactive agents (responding directly to stimuli) to more complex deliberative or learning agents that maintain internal models and plan ahead. A simple classification scheme has been proposed for AI agents and is summarised in Table 1 [19, 35, 41].

**Table 1:** An overview of different types of agents.

Type of agent	Description	Examples
Simple reflex	Operate on condition‐action or if‐then rules without memory. Simple, fast, widely used.	An alert system on a smart cast that immediately notifies staff if a preset temperature threshold (indicating potential infection or pressure issues) is crossed and recommends further treatment.
Model‐based reflex	Exist in an environment, abide by rules, and consider previous perceptions from their environment	A gait analysis sensor that flags deviations from a patient's established baseline walking pattern stored in its memory and instructs therapists and patients.
Goal‐based	Plan and execute to achieve specified goals. Always choose the optimal path	A navigation system used during joint replacement surgery that dynamically guides the surgeon to achieve the preplanned implant position goal, despite changes in the environment.
Utility‐based	Uses a utility function to choose actions to maximise expected utility, balancing conflicting goals	Software recommending the optimal rehabilitation protocol by weighing factors like predicted recovery speed, cost, patient tolerance and desired functional outcome utility.
Learning	Continuously improve strategies based on experience, becoming more autonomous. Can be utility‐ or goal‐based.	An artificial intelligence (AI) tool analysing patient‐reported outcome measures and therapy logs over time to continuously refine personalised physical therapy recommendations.
Problem‐solving	Employ search algorithms to achieve desired outcomes.	Preoperative planning software using search algorithms to determine the optimal sequence of corrective osteotomies for complex limb deformity correction.

## CORE COMPONENTS OF AN AI AGENT

**Figure 1:**
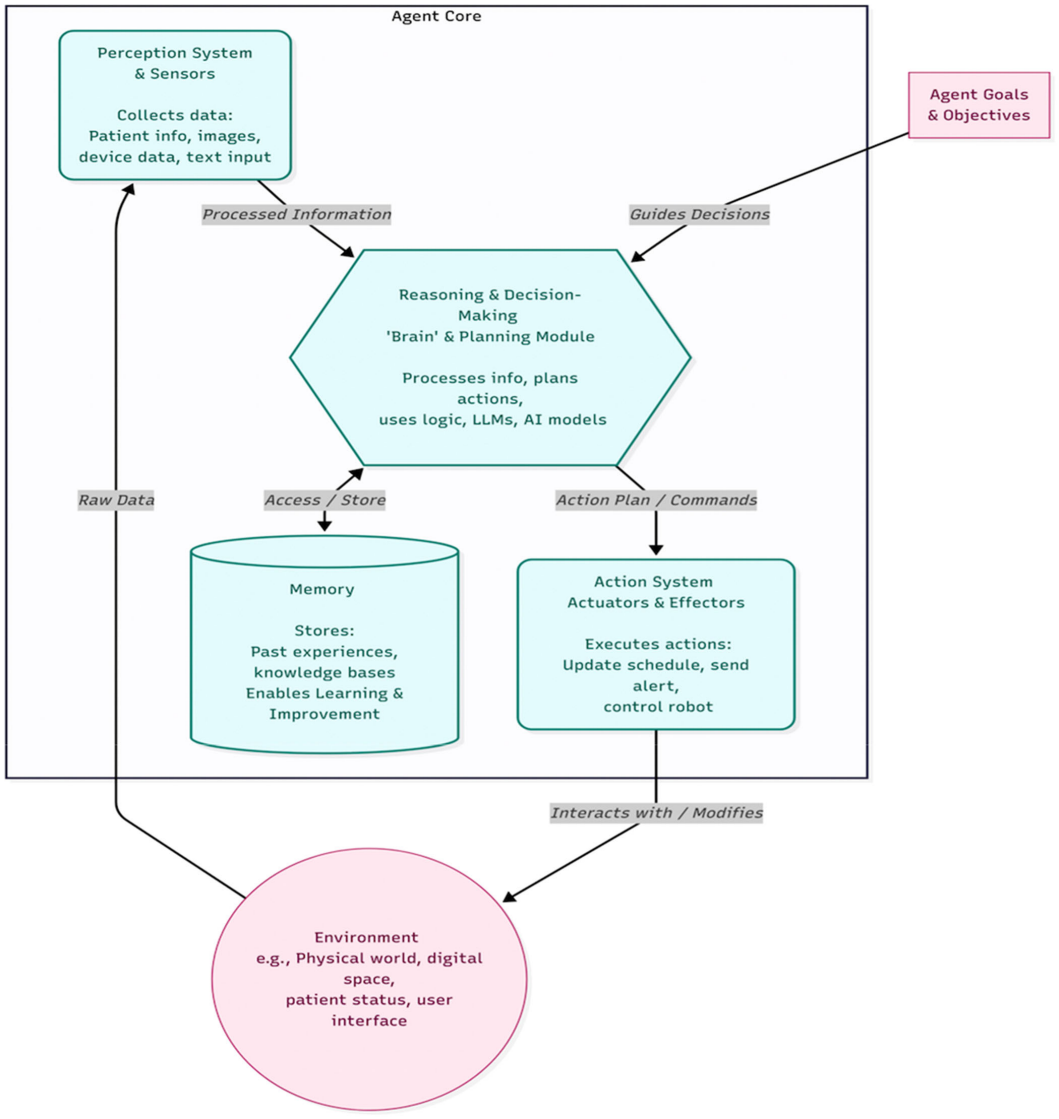
Sample Architecture of an artificial intelligence (AI) agent. This figure illustrates the core components of a typical AI agent. The perception system gathers raw data from the environment (e.g., patient information, sensor readings). This data are processed and sent to the reasoning & decision‐making engine (the ‘Brain’), which uses logic and AI models to create an action plan based on the agent's overarching goals and objectives. The engine accesses memory (a knowledge base of past experiences) to improve its decisions. The resulting action plan is sent to the action system, which uses effectors to interact with and modify the external environment (e.g., updating a clinical schedule or controlling a device). This entire process forms a continuous loop, allowing the agent to learn and adapt.

The agent's core requires inputs in order to understand, plan and execute complex tasks. Perception system and sensors collect data from the environment (e.g., patient data, medical images, real‐time device data, text input) [11]. This information is then processed and forwarded to the central decision‐making engine. The decision‐making engine responsible for reasoning and planning might be called the ‘brain’ of the agent. It processes information, uses logic, accesses knowledge bases (memory), plans actions and makes decisions to achieve goals, which often involves LLMs, RL, or other AI models [11, 22]. The memory of an agent is an important module as it stores past experiences and knowledge to learn and improve performance over time. The interaction with the environment is then facilitated through the action system driven by effectors. This module executes the decision into an action to interact with the environment (e.g., updating a schedule, sending an alert, controlling a robotic arm) [11, 22] (Figure 1).

**Figure 2:**
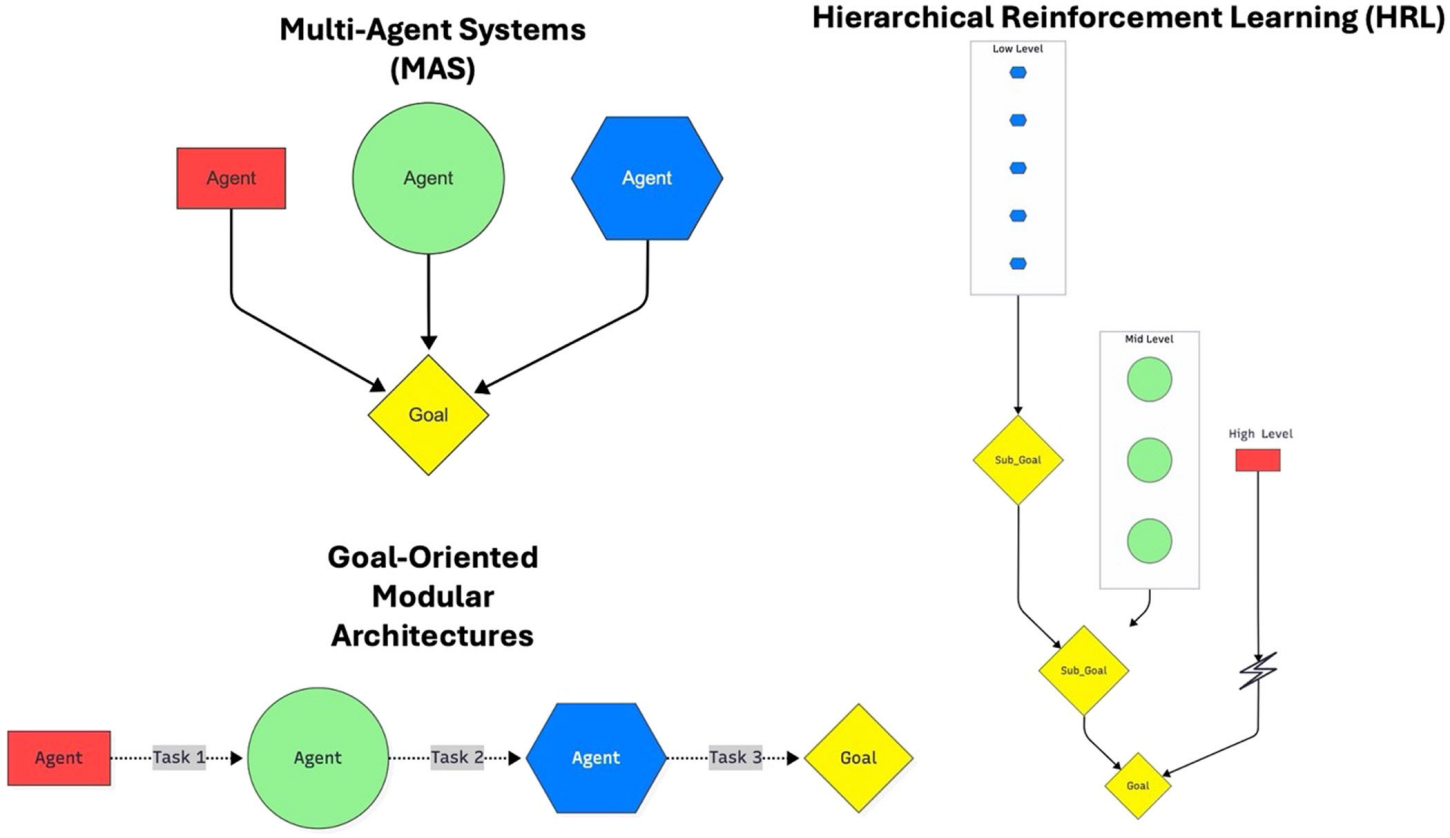
Illustration of three common architectures for implementing agentic artificial intelligence (AI). Multiagent systems (MAS) use multiple autonomous agents working towards a shared goal. Hierarchical reinforcement learning (HRL) structures decision‐making into levels, where higher levels set subgoals for lower levels. Goal‐oriented modular architectures divide a process into specialised modules, each responsible for a specific part of the task.

High agentic behaviour (autonomy) necessitates high trust. This trust is better supported by a robust agentic implementation that allows for explainability, predictability and validation [34]. Common architectural approaches for implementing agentic AI include multiagent systems (MAS), hierarchical reinforcement learning (HRL) and goal‐oriented modular architectures (Figure 2) [1]. In MAS, tasks are divided amongst multiple autonomous agents with a common goal [3]. In contrast, decision making is structured hierarchically in HRL, where high‐level agents define subgoals, which are executed by low‐level agents [39]. In goal‐oriented modular architectures, agent functions are modular, where each module specialises in specific aspects of a task [10]. These implementations can help enhance transparency, as interactions between components or agents are often structured and observable.

## CLINICAL APPLICATIONS AND FUTURE DIRECTIONS OF AGENTIC AI IN ORTHOPAEDICS

### Perioperative workflow optimisation

Perioperative care represents one of the most promising applications for AI agents in orthopaedics, where these systems can coordinate and optimise complex workflows across clinical management pathways. Through the analysis of historical case durations, surgeon availability and patient needs, scheduling optimisation AI agents can reduce delays, prevent overbooking and maximise operating room utilisation—addressing a critical inefficiency particularly relevant in orthopaedics, where procedures vary dramatically in duration and resource requirements [11, 15, 34]. The complexity of these systems increases significantly in high‐acuity settings such as trauma services, where hierarchical decision‐making processes must accommodate rapid changes to OR schedules, prioritise emergent cases and redistribute resources in real‐time based on patient condition and surgical urgency. Practical implementations are emerging through MAS dedicated to each phase of the perioperative process: preoperative, intraoperative and postoperative. A preoperative MAS might incorporate multiple specialised agents, including a Risk Stratification Agent utilising predictive analytics to assess surgical risks, a scheduling optimisation agent applying ML to maximise resource efficiency—including interdepartmental OR sharing and a preoperative communication agent facilitating seamless information flow between patients, surgical teams and administrative staff [22, 34]. These specialised agents may operate under the coordination of a preoperative master orchestrator agent that integrates data inputs and optimises decision‐making for surgical preparation. Similar multigent architectures for intraoperative and postoperative phases create a comprehensive system spanning the entire care continuum, potentially transforming efficiency and outcomes in orthopaedic surgery through intelligent automation and coordination [4, 22].

## RESEARCH ACCELERATION

AI agents may soon be capable of transforming research across various domains, however, they still lack strategic, purposeful implementation [9, 11, 13]. The literature demonstrates that these systems can autonomously search, read and synthesise thousands of studies—moving beyond simple keyword searching to understanding conceptual relationships and clinical implications, thereby identifying patterns, contradictions and knowledge gaps with unprecedented efficiency [9, 11]. In experimental design and optimisation, AI agents could recommend optimal methodologies, sample sizes and measurement approaches for biomechanical studies, clinical trials, or basic science experiments based on specific research questions and available resources [9, 12, 22]. In data analysis and interpretation, these agents can assist in both traditional statistical approaches, while also identifying complex patterns in research data, suggesting novel hypotheses and even generating preliminary manuscripts based on findings [12]. These capabilities represent a significant advancement over current research tools, potentially accelerating both the pace of discovery and clinical translation in orthopaedics by automating labour and time‐intensive tasks while uncovering insights that might otherwise remain hidden within the growing volume of scientific literature and research data.

## INTELLIGENT CLINICAL AND SURGICAL SUPPORT

The future landscape of agentic AI in orthopaedics likely extends beyond what is currently envisioned, with numerous applications still to be discovered. Among the more apparent opportunities on the horizon are intelligent assistant agents which could function as copilots for healthcare providers, reducing cognitive burden and administrative workload by retrieving information, drafting documentation, managing alerts, summarising patient histories and facilitating routine patient communications [11, 34]. These systems will evolve alongside advanced decision support tools that integrate comprehensive datasets spanning radiomics, wearable technology and outcomes metrics to deliver increasingly personalised predictions regarding implant success and complication risks in both perioperative and outpatient contexts [11].

Further developments are anticipated in patient management optimisation, with AI agents intelligently prioritising cases based on acuity, complexity and resource availability while dynamically adjusting clinical and surgical schedules to enhance efficiency without compromising care quality [11, 34]. Real‐time procedural guidance represents another frontier, particularly in surgical settings where hybrid AI models may provide intraoperative decision support [11, 22, 34]. Additionally, remote monitoring capabilities will expand through AI systems that continuously analyse data from wearable devices and patient‐reported outcomes, identifying concerning trends and coordinating interventions before complications manifest, thereby extending the reach of orthopaedic care beyond traditional clinical boundaries.

## THE CONTINUUM OF SOFTWARE AND ROBOTIC AGENTS

The landscape of agentic AI encompasses diverse implementation approaches, with software‐based agents representing the major category. These digital entities analyse clinical data, generate recommendations and streamline workflows without physical intervention capabilities [11, 34, 50]. Their digital nature enables rapid deployment and iteration, making them useful for clinical decision support systems, research assistance tools and administrative process optimisation. In contrast, robotic systems with agentic components may merge computational intelligence with physical capabilities, improving on largely passive surgical robots to such that will precisely execute surgeon commands to more advanced systems which will be capable of adapting to changing conditions during procedures. Rather than representing distinct categories, these implementations will exist along a continuum where many promising applications combine elements of both—resulting in intelligent software agents that enhance planning for robotic procedures or increasingly autonomous robotic systems guided by sophisticated agentic software.

The healthcare field has much to gain from MAS, with applications emerging across diagnostics, collaborative decision support, prehospital emergency response and automated insurance justifications [4, 12, 34]. These systems feature multiple specialised agents working in concert through collaboration or negotiation protocols [4, 11, 34]. Notable examples include MD Agents, which leverages large language models within a multiagent framework for medical decision‐making and systems applying the MAS approach to modular healthcare data analysis [21]. Such architectures may simulate healthcare team collaboration, analyse complex data streams and perform other tasks requiring distributed intelligence—pointing toward future systems where specialised digital agents collaborate much like human healthcare teams to deliver comprehensive orthopaedic care.

## IMPLEMENTATION CONSIDERATIONS

The implementation of agentic AI in orthopaedics faces numerous challenges, not entirely unique compared to other medical specialties and also other industries. Seamless integration with existing electronic health records (EHRs) and imaging systems remains an initial hurdle, as these agents must function within established clinical workflows. Data quality presents another obstacle—as up to 80% of the EHR consists of unstructured data [20, 28], which is prone to human error [43, 47]. This necessitates either algorithms capable of standardising diverse inputs or a transition to more compatible information systems. Additionally, these applications must achieve real‐time performance with minimal latency while processing complex, multimodal data streams, not only for intraoperative use cases, but already in administrative tasks, as multiple patients might be requesting help simultaneously.

Beyond these technical considerations, the successful deployment of AI agents in orthopaedics depends on establishing appropriate validation metrics and explainability for clinical performance—a critical step for both regulatory approval and physician confidence [32, 33]. Economic factors will inevitably influence adoption rates, as practices must weigh implementation costs against potential efficiency gains and improved outcomes. Accordingly, evaluation of these agents, currently limited to accuracy/task completion metrics, should include cost‐effectiveness measures to facilitate practical deployment in resource‐limited environments [17]. Perhaps most importantly, these systems must earn acceptance from both patients and healthcare providers, who may harbour reservations about AI agents. As the field progresses, addressing these implementation barriers will be essential for realising the full potential of agentic AI in orthopaedic practice.

## ETHICAL AND LEGAL FRAMEWORK

The ethical and legal landscape surrounding agentic AI in orthopaedics presents significant challenges. Regulatory frameworks remain underdeveloped, with bodies like the US Food and Drug Administration (FDA) and European Medicines Agency (EMA) still establishing pathways for evaluating adaptive AI technologies [48]. This uncertainty is compounded by complex questions of liability. When an autonomous agent contributes to an adverse outcome, determining responsibility—whether it lies with the developer, the healthcare provider, or the institution—becomes legally and ethically fraught, particularly when the agent's decision‐making process is not fully transparent.

Privacy and data governance represent another critical dimension. Agents that continuously learn from clinical data challenge traditional notions of patient consent and confidentiality, while the immense volumes of sensitive data they require raise questions about storage and access. Furthermore, equity considerations loom large. There is an inherent risk that biases present in training data (e.g., from specific patient demographics) could be amplified, exacerbating health disparities. Similarly, these advanced technologies might become disproportionately available in well‐resourced settings, widening the gap in care quality. Addressing these challenges requires thoughtful collaboration between clinicians, scientists, ethicists and policymakers to ensure that the benefits of AI are realised responsibly.

## CONCLUSION

AI agents, representing a generational improvement upon traditional AI, present a potentially practice‐changing shift in orthopaedic practice. While promising applications are already possible in perioperative optimisation, research acceleration and workflow management, implementation faces significant technical, ethical and regulatory challenges. As orthopaedics navigates this frontier, success will depend on thoughtful integration that augments, rather than replaces, human expertise. For the orthopaedic surgeon, engaging with these technologies is no longer a niche interest but a professional necessity. Understanding the principles of agentic AI will be crucial for leading clinical teams, shaping the future of surgical practice and ensuring that these powerful tools are implemented safely and effectively to the ultimate benefit of the patient.

## AUTHOR CONTRIBUTIONS

All listed authors have contributed substantially to this work. F.C.O., J.P. and B.Z. performed literature review. F.C.O. performed primary manuscript preparation. Editing and final manuscript preparation were performed by J.P., B.Z., Y.Y., T.C., R.F., P.W.W., M.T.H. and K.S. All authors read and approved the final manuscript.

## CONFLICTS OF INTEREST STATEMENT

Prof. Kristian Samuelsson is a board member of Getinge AB. The remaining authors declare no conflict of interest.

## ETHICS STATEMENT

The authors have nothing to report.

## Data Availability

Data sharing is not applicable to this article as no datasets were generated or analysed during the current study.
